# Genetic Relatedness and Parentage Analysis as a Framework to Enhance Local Conservation Strategies for Marine Species

**DOI:** 10.1002/ece3.72184

**Published:** 2025-09-16

**Authors:** Thomas Guttierez, Emilie Boissin, Serge Planes

**Affiliations:** ^1^ PSL Research University, CNRS‐EPHE‐UPVD, UAR3278 CRIOBE Perpignan France; ^2^ Laboratoire d'Excellence “Corail” Perpignan France

**Keywords:** *Atrina vexillum*, Bora‐Bora, genetic relatedness, marine conservation, parentage analysis, UPGMA clustering

## Abstract

Conservation of marine species faces specific challenges, especially considering the environmental factors and cryptic behaviors that affect organismal dispersal. In the context of conservation genetics, microsatellite markers have been successfully applied in population genetics to assess genetic differentiation and delineate population clusters, helping to identify broader scale conservation needs. Here, we tested the relevance of using microsatellite markers to perform parentage analysis with very little information on a marine sessile organism to inform spatial conservation efforts. Specifically, we investigated the genetic structure of *Atrina vexillum*, a large marine bivalve, sampled in the lagoon of Bora‐Bora. Our primary objective was to sample a significant portion of the population with a stratified underwater survey, which led to the sampling of 1389 individuals, estimated to represent ~80% of the total population. We then screened 30 microsatellite loci to compute a relatedness matrix and assess genetic relationships among individuals. Based on pairwise relatedness values, we computed a UPGMA hierarchical clustering to construct family groups and compared the results with a maximum‐likelihood‐based parentage analysis implemented in COLONY. In parallel, we also looked for genetic structure using Bayesian clustering with STRUCTURE. We then investigated spatial patterns of genetic relatedness, testing correlations with geographic distances. The distribution of relatedness values indicated a relatively even contribution to reproduction among sampled individuals. Although no population structure was detected using the Bayesian clustering algorithm, we identified multiple family groups, some comprising up to 19 individuals. Spatial analyses showed no significant correlation between genetic relatedness and proximity, except among closely related individuals, who tended to be found in close proximity. Our findings highlight the value of the relatedness‐based approach to identify genetic connectivity and reproductive behavior. In *A. vexillum* , this approach demonstrates the importance of protecting high‐density areas where settlement occurs, even though no spatial genetic structure was found within the lagoon.

## Graphical abstract

We investigated the genetic structure and parentage relationships within a nearly complete population of *Atrina vexillum* in Bora‐Bora. Using 30 microsatellite markers and spatially explicit sampling, we found strong family clustering despite the absence of overall population structure. Our findings highlight the value of relatedness‐based UPGMA clustering to inform local conservation strategies in lagoon ecosystems.

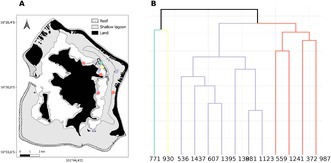

## 1 Introduction

The growing exploitation of natural resources, combined with rising pollution and global warming, highlights the need to refine species conservation strategies to preserve biodiversity and maintain ecosystem resilience. Effective conservation efforts face two major challenges: economic constraints and technical limits. To optimize conservation efforts, management units are often delineated based on administrative or geographical boundaries (Funk et al. 2012). While this approach can be effective, neglecting genetic connectivity may lead to an inaccurate definition of conservation scales and required efforts. Reproductive success, a key target of conservation, is not uniformly distributed across space (Hedgecock and Pudovkin 2011), and failing to account for this heterogeneity may compromise the effectiveness of targeted protections.

The marine environment presents specific challenges, with the vast majority of organisms going through a planktonic phase during their life cycle, making it difficult to define geographical boundaries that are not only dependent on the benthic topography but also a function of many physical and biological conditions such as currents, temperature, or buoyancy behavior (Cowen et al. 2006; Shanks 2009). In this context, reproductive success and its variability are more difficult to identify and remain largely understudied in the literature.

Sessile species in marine environments have reproduction and dispersal mechanisms that make them sensitive to local perturbations. Their inability to move toward optimal environmental conditions once settled makes the reproduction and planktonic phases the ultimate ways to ensure the stability and renewal of the populations. Therefore, understanding the reproductive dynamics, connectivity, and life history (Baguette et al. 2013; Montero‐Serra et al. 2018; Pineda et al. 2007) of these species is one of the main challenges we face when aiming to protect a population. Indirect approaches using genetic markers have formed the foundation of the concept of conservation genetics that will provide key information on the effectiveness of individuals' reproduction, even with undocumented reproduction data performed by broadcast spawners (Vendrami et al. 2021). By targeting protection on specific individuals showing the highest effective reproduction, it becomes possible to apply a policy of concentrating resources and temporarily halting or reversing (Gobler et al. 2022) population loss. For marine organisms, genetic connectivity is used as a proxy for marine population connectivity to demonstrate isolation by distance at a large scale (Selkoe and Toonen 2011) and guide management plans (Palumbi 2003; Suck An et al. 2011). It reflects the dynamics of the actively reproducing individuals. Among the diverse markers used to assess the connectivity of bivalves, simple sequence repeats (SSRs, also called microsatellites) have shown genetic variability sufficient to detect local allele frequency variation within bivalve populations (Rose et al. 2006). The dispersal modality of bivalves occurring in the fecundation and larval phases usually ensures a homogeneous genetic pool within populations of fan mussels in the same geographic region (Peyran et al. 2021; Wesselmann et al. 2018; Zhu et al. 2024).

Bivalves, together with corals and calcareous algae, are the engineering species of the reef. Their presence may affect communities by clarifying (Peterson and Jr 2001) or supporting hard‐substrate‐dependent (Lacoste et al. 2016; Lacoste and Gaertner‐Mazouni 2015; McKinney 1996; Norling et al. 2015). They are facing multiple pressures that lead to an overall decline, including overexploitation (Huang et al. 2023), marine heat (Masanja et al. 2023), ocean (Kruft Welton et al. 2024), pollution or anthropization (Mayer‐Pinto et al. 2020), and introduction of invasive (Diga et al. 2022). *Atrina vexillum* (Born, 1778) is an Indo‐Pacific fan mussel facing those problematics. Present in French Polynesia, it is characterized by a large size, reaching up to 48 cm in length (Poutiers 1998). The reproductive system of *A. vexillum* has not been formally described; spawning activity has been reported in the coastal waters of southern Vietnam, with mature individuals observed during March and April (Malakhov et al. 1985). However, these observations were made under different environmental conditions, and no definitive conclusion can be drawn regarding the species' reproductive mode (e.g., gonochorism vs. hermaphroditism), which remains undocumented. Among closely related pinnids, *Atrina maura* is gonochoric (Camacho‐Mondragón et al. 2012), whereas *Pinna nobilis* exhibits successive (protandrous) hermaphroditism (De Gaulejac 1995).

Concerned by its unquantified population decrease, the local government of French Polynesia placed the species under the status of strictly protected species, while it has been historically fished in the Indo‐Pacific region (Rosewater 1961). *A. vexillum* has been understudied despite its economic importance and is the subject of very few studies (Huang et al. 2015; Lemer et al. 2014; Ning et al. 2016; Reid and Brand 1989; Silina 2012), and none are related to the conservation of the species. A recent study on *A. vexillum* has highlighted a possible differentiation between islands separated by 30 km (Guttierez et al. 2025), which may reflect a unique dispersal dynamic and conservation challenge.

In this study, we aim to investigate how genetic markers like SSRs can reveal patterns of reproductive contribution among individuals for an isolated marine population at the scale of a single small island, Bora‐Bora (French Polynesia). Using a set of 30 newly developed microsatellite markers (Guttierez et al. 2025), we investigate the relevance of population genetics in defining a conservation strategy for a marine sessile species. Exploring population structure by detecting clusters of individuals with similar allele frequencies is one way to achieve this goal and is commonly used (Reisser et al. 2019; Wei et al. 2023; Xu et al. 2020). Relatedness at the individual level is less investigated because marine species, due to their planktonic phase, have a wider scale of dispersal and require a larger sampling effort to refine related individuals (D'Aloia et al. 2015), but it is crucial to investigate how reproductive success is spatially distributed. Finally, family structures have rarely been reported in marine species (Cui et al. 2024; Morvezen et al. 2013; Nebot‐Colomer et al. 2022; Peyran et al. 2022), and would explain how dispersion occurs over a generation.

We focused on three main objectives. First, we aimed to characterize the population of *A. vexillum* in Bora‐Bora through a nearly exhaustive and the analysis of genetic diversity. Second, we assessed pairwise relatedness among individuals and produced a list of family clusters using UPGMA, compared to the COLONY‐based kinship estimation. Finally, we explored spatial patterns of genetic relatedness and the heterogeneity in reproductive success with the goal of providing critical information on priority areas for conservation of the island population.

## 2 Materials and Methods

### 2.1 Sampling Strategy

In 2022, a detailed survey on *A. vexillum* was conducted in Bora‐Bora (16°29′35″ S, 151°44′12″ W) over a period of approximately 6 weeks during May and June 2022, leading to the sampling of 1389 individuals. The sampling was designed to be as exhaustive as possible. Based on direct field observations, repeated site visits, and a combination of survey methods targeting all identified suitable habitats across the lagoon, we estimate that our sampling covers approximately 80% of the total *A. vexillum* population in Bora‐Bora.

Tissue samples for DNA extraction were collected using a minimally invasive method transposed from (Peyran et al. 2022). Forceps were used to gently remove a small piece of tissue from the edge of the mantle, which is typically exposed when the valves are slightly open. This nonlethal procedure minimizes harm and stress to the individuals, thereby preserving their viability after sampling.

As snorkeling is the most efficient way to cover a large area with a complex benthic topology and/or high population density in shallow waters (up to 15 m), two free divers selected a geographically identifiable area from the surface and split it into two sections while maintaining visual contact to ensure safety. They individually surveyed their respective areas to avoid double sampling.

For less complex areas, we used the towed free diver technique. A free diver was towed at low speed (~1–2 knots) behind, allowing systematic visual detection of individuals on the lagoon floor (mostly sand or mud).

Finally, for individuals settled below 15 m, we deployed scuba divers. The area of exploration was restricted to either large spaces between 15 and 20 m (though few such areas met the necessary conditions) or to shallower depths with small areas that had a specific focus on artificial submerged structures.

The total sampling effort was 0.37 km^2^ for snorkeling, 3.12 km^2^ for towed free diving, and 1.23 km^2^ for scuba diving. All spatial visualizations, including the distribution of sampled individuals and survey coverage, were generated using QGIS v.3.34.11‐Prizren. While accounting for a total of about 5 km^2^ surveyed in detail within the 165 km^2^ lagoon of Bora‐Bora, this surface corresponds to what we identified as the essential habitat of the species.

In order to carry out a first genetic study on the species, all individuals were sampled with a non‐lethal method previously elaborated by Peyran et al. (2022) on large bivalves, and small tissue samples were preserved in absolute ethanol. The width of the individuals was measured at the largest measurable section outside the substrate, and their sampling depth was recorded. Size differences stratified into depth classes of 5 m were tested by performing a Kruskal–Wallis test, a nonparametric alternative to ANOVA followed by a post hoc Dunn's test, adjusted with a Bonferroni correction.

### 2.2 DNA Extraction and Amplification

In order to extract Genomic DNA from each individual, the QIAamp 96 DNA QIAcube HT Kit (QIAGEN) was used according to the manufacturer's instructions. Some samples presented a low quantity of tissues (*n* = 64). They were extracted separately using the Gentra Puregene Tissue kit (QIAGEN). Following the same methodology and the microsatellites developed by Guttierez et al. (2025), five mixes of six SSRs each were performed. The 30 microsatellites were composed of 14 di‐nucleotides, seven trinucleotides, and nine tetranucleotides markers included in the five multiplex. Multiplexed SSRs have been proved reliable for parentage analysis in other bivalves (Morvezen et al. 2013; Peyran et al. 2022). The amplicon was then sent to GenoScreen for capillary electrophoresis using an Applied Biosystems 3730xl Genetic Analyzer (Thermo Fisher).

The alleles were scored using GENEMAPPER software v.5 (Applied Biosystems). To avoid any reading bias, individuals were examined independently of the PCR reaction order. During this process, any uncertain peak lengths or shapes were excluded and marked as missing data. The minimal amplification success rate per individual was set to 75%. Following these criteria, 1205 individuals out of the 1389 sampled were retained for analysis.

### 2.3 Genetic Diversity and Population Structure

#### 2.3.1 Genetic Diversity Metrics

GenAlex v.6.5 (Peakall and Smouse 2006, 2012) was used for genetic diversity analysis, including the number of alleles per locus (*N*_a_), observed (*H*_O_) and expected (*H*_E_) heterozygosities per locus. Inbreeding coefficients (*F*_IS_) were calculated using GENETIX v 4.05.2 (Belkhir et al. 2004). Significance was corrected using the sequential Bonferroni correction in R (R Core Team 2021) with the ‘p.adjust’ function for multiple testing.

The microsatellite markers used in this study were selected from the 30 loci developed by Guttierez et al. (2025).

#### 2.3.2 Population Structure Analysis

To first determine whether groups of individuals with similar allele frequencies might exist, we performed Bayesian clustering using STRUCTURE (Pritchard et al. 2000) software, with an admixture model and correlated allele frequencies between putative clusters. The number of populations (*K*) varied from 1 to 10, and for each value of *K*, 10 independent replicates were run with 100,000 burn‐in iterations followed by 1,000,000 Markov chain Monte Carlo simulations. The optimal number of clusters (*K*) was determined using the Puechmaille (2016) method with four estimators (MedMedK, MedMeaK, MaxMedK, and MaxMeaK), which makes fewer assumptions about the relative cluster size distribution than the Evanno et al. (2005) method. This approach was chosen due to the limited ecological data available on the distribution of *A. vexillum* in French Polynesia and the potential uneven sampling. To provide a representation of this cluster attribution and the different metrics used by Puechmaille, we used Structure Selector (Li and Liu 2018).

### 2.4 Relatedness and Family Identification

Without information about the individuals sampled, such as their age or sex, it is not possible to determine the direction of gene transmission. However, it is possible to estimate the degree of kinship (Blouin 2003). We examined the family structure among the individuals sampled in Bora‐Bora using the software ML‐RELATE (Kalinowski et al. 2006) that calculates the maximum likelihood estimates of relatedness (r) between individuals with a bias‐reducing method regarding null alleles (Wagner et al. 2006). Pairs that demonstrated an r>0.95 (*n* = 51) were considered as duplicate samples, and only one of the samples was kept for the rest of the analysis.

From the obtained individual pairwise relatedness matrix, a distance matrix (1−r) was calculated and used to construct a UPGMA dendrogram using the ‘hclust’ function from the stats package in R (R Core Team 2021). This hierarchical clustering formed nodes representing the fusion of two clusters, which we chose to evaluate using a central estimator by calculating the mean $\left(\bar{r}\right)$ of all r among subsequent individuals (Equation 1).

(1) $${\bar{r}}_{N}=\frac{1}{n_{1}n_{2}}\sum_{I_{i}\in C_{1}}\sum_{I_{j}\in C_{2}}r\left(I_{i},I_{j}\right)$$

with N a node merging two clusters $C_{1}$ and $C_{2}$ of sizes $n_{1}$ and $n_{2}$, respectively, where $I_{i}$ and $I_{j}$ are individuals in $C_{1}$ and $C_{2}$, ${\bar{r}}_{N}$ represents their mean pairwise relatedness coefficient.

We have set a ${\bar{r}}_{N}$ threshold at 0.25, corresponding to a half‐sibling theoretical kinship, that would delimit a cluster with closely related individuals. Only the largest clusters possible meeting this requirement, independently of the subsequent node, were considered, providing an assemblage of family entities.

To corroborate the family structure characterized above, we tested a Bayesian approach based on maximum likelihood analysis implemented in the software COLONY (Jones and Wang 2010) also used for parentage analysis (Harrison et al. 2013). Parameters were set as polygamy for female and male individuals, without clones, under the dioecious assumption, following a high likelihood precision for a medium run length. COLONY was also used to estimates the effective population size ($N_{e}$) with full likelihood method (Wang 2004) under the random mating assumption, since no information about the species might refer to anti‐inbreeding mechanisms.

### 2.5 Spatial Distribution of Genetic Relatedness

To investigate the relationship between genetic relatedness values among individuals and their spatial distributions, we tested the variation in pairwise geographic distances between individuals. To do so, we grouped individual pairs into relatedness classes, defined by increasing levels of pairwise relatedness. These classes reflect the degree of genetic similarity between individuals, estimated from multilocus genotypes, and are used to explore patterns of spatial clustering among relatives. To avoid an unbalanced sample sizes between classes as high relatedness values are relatively rare in natural populations, we applied a bootstrapping procedure (*R* = 1000) to resample each relatedness class to the size of the least represented category. This resampling makes the result more reliable against unequal sample size differences.

For each bootstrap iteration, we performed a Kruskal‐Wallis test to assess whether geographic distances differed significantly among relatedness classes. The stability of the results was evaluated by the median *p* value across all iterations, along with a 95% confidence interval for the distribution of *p* values obtained through bootstrapping. Statistical significance was considered robust as long as the median *p* value remained below 0.05 and if the confidence interval did not overlap this threshold. If the global Kruskal‐Wallis test was significant, we conducted a post hoc Dunn's test, adjusted with a Bonferroni correction to control for multiple comparisons. This test identified which pairs of relatedness classes exhibited significant differences in pairwise distances. In other words, we tested whether related individuals were distributed more closely together spatially.

## 3 Results

### 3.1 Bora‐Bora Survey

**FIGURE 1:**
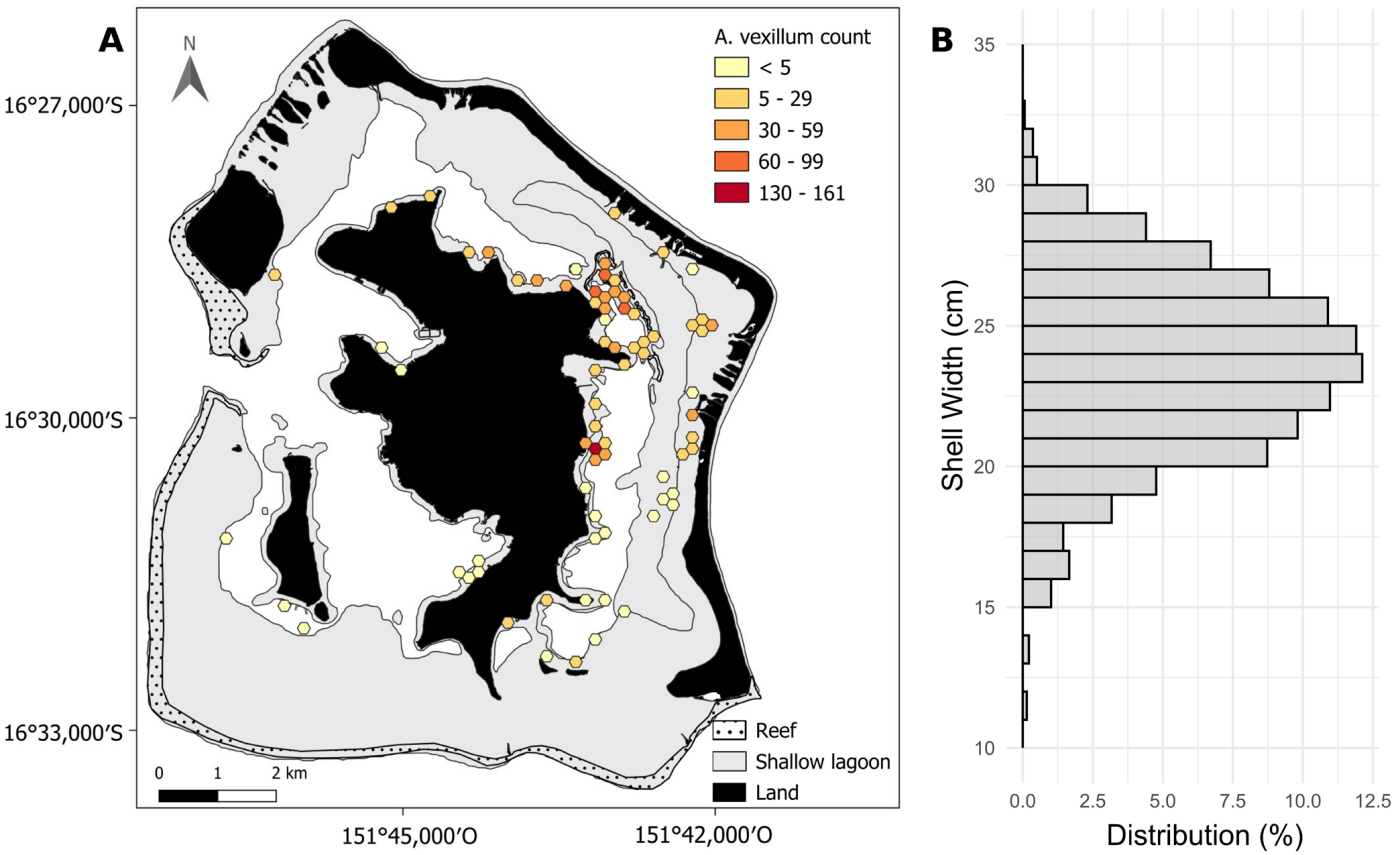
Spatial distribution and size structure of *Atrina vexillum* in the lagoon of Bora Bora. (A) Density of individuals observed with a 3.46‐ha hexagonal grid. (B) Distribution of the shell width measured for each sampled individual.

Samples of *A. vexillum* collected around the lagoon of Bora‐Bora showed a heterogeneous distribution, as 97% of the individuals were collected in the eastern part of the lagoon with a higher concentration in the northern areas (Figure 1A). The mean depth at which shells were sampled was 4.9 m (± 7.4 SD), with a maximum depth of 26 m. The size distribution, based on the largest shell width measured for each individual (mean 24.1 cm ± 3.2 SD), showed a unimodal distribution, with most individuals centered around 25 cm (Figure 1B). Shell width distributions varied significantly among 5 m depth classes (Kruskal–Wallis; *p* < 0.001). Post hoc tests identified the difference between [0–5] (meters class [mean = 23.4 cm ± 3.1 SD width]) with [5–10] and [25–30] meters classes (respectively mean = 24.3 cm ± 2.8 SD and 26.5 cm ± 2.3 SD width) (Dunn test; *p* < 0.01), indicating that individuals sampled at shallower depths tend to be smaller than the ones sampled deeper.

### 3.2 Genetic Diversity of *Atrina vexillum* in Bora‐Bora

The 21 SSR markers provided a high level of genetic diversity as indicated by the number of alleles per locus (mean = 9.9 ± 5.0 SD), ranging from 4 to 21. The analyzed individuals showed a mean expected heterozygosity (He) of 0.67 ± 0.18 (SD) and a similar mean observed heterozygosity (Ho) of 0.65 ± 0.17 (SD) per locus, ranging from 0.29 to 0.91 and 0.28 to 0.91, respectively (Table S1). Moderate significant deviations from Hardy–Weinberg equilibrium were observed for eight loci. Overall, the population exhibited moderate levels of inbreeding (*F*_IS_ = 0.02, *p* < 0.01).

Assuming random mating as the primary test, the COLONY full likelihood method estimated an effective population size (*N*_e_) of 902 individuals (Confidence interval of 95%: [814–994]), representing 75% of the 1205 individuals considered for these genetic analyses.

### 3.3 Population Structure Analysis

Genetic differentiation evaluated by STRUCTURE showed a high level of admixture for all values of *K* tested and identified according to Puechmaille's method (Puechmaille 2016), a single cluster over the entire sampling area (Figure S1A,B). Although multiple sub‐clusters (*K* = 5) were suggested by the Evanno's method (Evanno et al. 2005), they could not be clearly distinguished (Figure S1C) given the high admixture, thus supporting the reliability of the Puechmaille's method.

### 3.4 Distribution of Relatedness Coefficients

**FIGURE 2:**
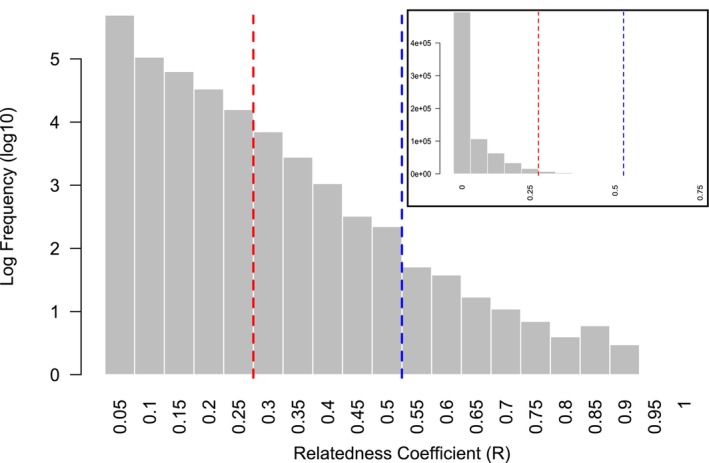
Distribution with log‐transformed frequency (log_10_) of observed relatedness values (*R*). Vertical dashed lines indicate theoretical kinship thresholds: *R* = 0.25 (red) for half‐siblings, and *R* = 0.5 (blue) for full siblings or parent‐offspring relationships. Pairs that demonstrated an r>0.95 were considered as duplicate samples. Inset shows the same distribution with raw frequency.

Considering all pairwise relationships among the analyzed individuals, the relatedness distribution followed a clear and constant decline under a decreasing exponential law usually observed in natural populations, with most individuals being unrelated and a fewer being related (Figure 2). No distinct peak was observed at the theoretical kinship threshold. Overall, the vast majority of individuals were not related or only slightly related. Of the 725,410 pairwise comparisons, only 1.59% showed a relatedness coefficient *R* > 0.25, and just 0.02% exceeded *R* > 0.5, confirming that close relationships were extremely rare in the dataset. Notably, only a few pairs displayed very high values of *R* > 0.75 (*n* = 10).

### 3.5 Clustering and Family Identification

**FIGURE 3:**
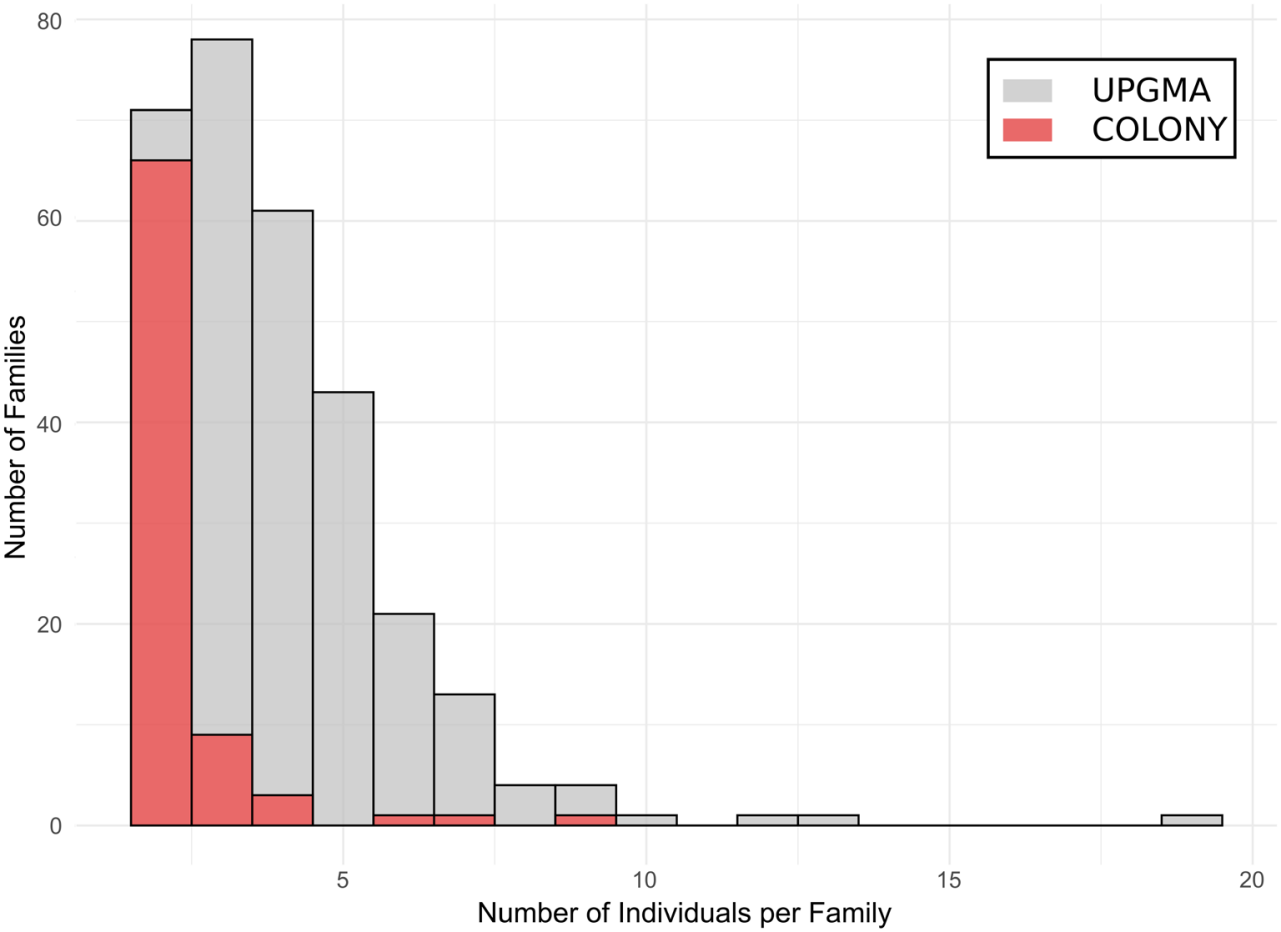
Histogram of family counts by number of individuals per cluster, based on UPGMA clustering in gray and COLONY inference overlaid in red.

Using the mean relatedness threshold of 0.25, the UPGMA clustering identified 299 family groups involving 97% of the analyzed individuals. The largest family size contained 19 individuals (mean 3.9 ± 2.0 SD; Figure 3), with a mean relatedness of 0.31. Over 66% of the individuals belonged to one of the 150 families with more than three members. Notably, several families had more than seven individuals, including four families of eight and nine individuals and four families of each one 10, 12, 13, and 19 members, respectively.

Using the same dataset, the COLONY modeling recovered fewer families (*n* = 156), representing only 13% of the individuals analyzed. Among the six families with more than three individuals recovered by COLONY, 97% of the individuals were grouped identically by UPGMA, and two of these families were merged by UPGMA clusters.

### 3.6 Influence of Relatedness on Spatial Distribution

**FIGURE 4:**
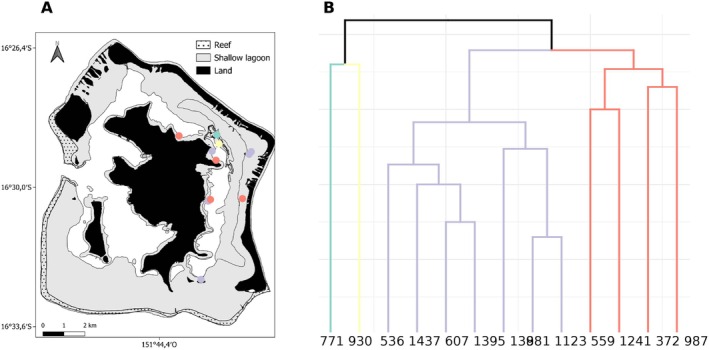
Example of the distribution of the individual across the lagoon of Bora‐Bora (A) colored by identified UPGMA (B) cluster of 2 (blue & yellow), 4 (red) and 7 (purple) members.

The spatial structuring of family groups in the Bora‐Bora lagoon showed no apparent spatial patterns (Figure 4A,B). The mean distance between individuals grouped by the UPGMA was not significantly different from the mean distance between randomly selected individuals (mean 2.2 ± 1.6 km SD).

**FIGURE 5:**
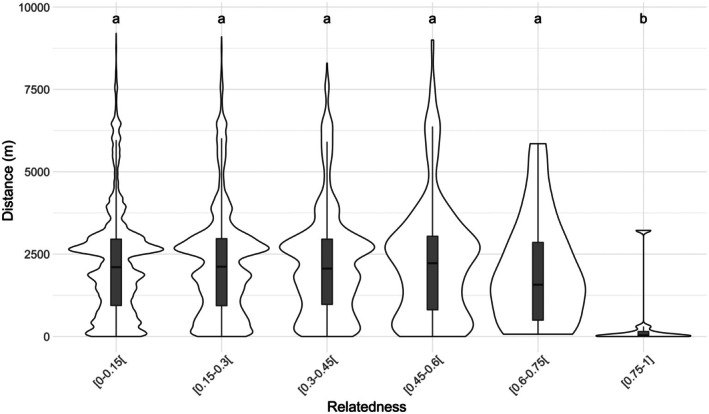
Violin plots of pairwise distances between individuals, with boxplots showing medians and interquartile ranges. Letters indicate statistical groups from Dunn's test (*p* < 0.001), where different letters denote significant differences.

When relatedness values were subdivided into classes, a significant effect on spatial distance was detected (Kruskal‐Wallis test; bootstrapped 95% CI for *p* value < 0.001, *R* = 1000). Dunn's test confirmed differences between the class *R* > 0.75 and all other classes (*p* < 0.001) (Figure 5). No other differences were observed among the other classes. The corresponding violin plot shows a notable decrease in average distance between individuals with relatedness values above 0.6, and especially stronger for relatedness values above 0.75.

## 4 Discussion

### 4.1 Key Findings

A single, genetically diverse population was identified among the 1389 *A. vexillum* individuals sampled from the Bora‐Bora lagoon. No population structure was detected by Bayesian clustering, and a high level of admixture was observed across all tested values of *K*, supporting the presence of a panmictic population. The absence of juveniles, based on shell width measurements, suggests that successful recruitment events are infrequent, leading to limited larval settlement in recent years.

The relatedness‐based approach revealed numerous family clusters, some including more than 10 individuals, as identified using UPGMA clustering. This structure was further supported by the COLONY analysis. The observed isolation‐by‐distance for high relatedness values suggests a locally restricted dispersal mechanism, leading to kin aggregation and the formation of spatially coherent groups of related individuals.

### 4.2 Skewed Demographic Structure and Low Recent Reproductive Success

The spatial distribution of *A. vexillum* within the Bora‐Bora lagoon was highly uneven, with 97% of individuals concentrated on the eastern side of the lagoon, particularly in the northern sector. This pronounced clustering likely reflects favorable environmental conditions, such as hydrodynamics, substrate type, and food availability, that enhance larval settlement or adult survival in this area. Similar aggregation patterns have been documented in other bivalves, where localized environmental conditions or hydrological retention zones promote larval recruitment (Barros et al. 2013; Kurihara et al. 2018).

The observed unimodal size distribution centered around 25 cm and the absence of smaller size classes suggest limited recent recruitment, which is consistent with the genetic data. In addition, the significant variation in shell size across depth classes suggests a depth‐related growth gradient, with larger individuals found at greater depths. This pattern may result from age stratification, reduced thermal or anthropogenic stress in deeper waters, or environmental factors influencing growth (Kurihara et al. 2018).

Based on the growth curve of *A. vexillum* , which allows individuals younger than 3 years old to be identified by the shell size (Silina 2012), we infer that no significant recruitment has occurred within the past 3 years. In many bivalve populations, natural variability in recruitment success is driven by environmental sensitivity during the larval and settlement stages (Vadopalas et al. 2012; Van Der Meer et al. 2001). For long‐lived and highly fecund species like *A. vexillum* , such variability may reflect some adaptive strategy, ensuring that each individual is likely to experience at least one successful reproductive event during its lifetime (Ripley and Caswell 2006).

Altogether, these spatial and depth‐associated patterns support a patchy and environmentally constrained recruitment dynamic within the lagoon. They reinforce the conclusion that the current population structure of *A. vexillum* is shaped by episodic reproductive success, localized settlement, and depth‐related survival processes, a pattern also observed in other bivalves such as *P. nobilis*, where limited larval dispersal and kin aggregation suggest highly localized and irregular recruitment (Nebot‐Colomer et al. 2022; Peyran et al. 2022), or in large bivalves like *Panopea generosa* , which also exhibit heterogeneous settlement patterns (Vadopalas et al. 2012).

### 4.3 A Single Genetically Diverse Population

The 21 SSR markers revealed a high level of genetic diversity as indicated by the mean number of alleles per locus (*n*_a_ = 9.9) together with high observed and expected heterozygosity values. The *F*_IS_ values per locus found in our study (ranging from 0 to 0.11) are notably low when compared to similar bivalves of the same region as *Pinctada margaritifera* (Lemer and Planes 2014) or *Tridacna maxima* (Laurent et al. 2002) with *F*_IS_ values of −0.05 to 0.53 and 0.18–0.24, respectively; and even lower when comparing more globally to bivalve datasets (Plough 2016; Zouros et al. 1988).

Although the Evanno method identified five clusters as the most likely scenario, a high level of individual admixture persisted consistently across all 10 tested values of K in STRUCTURE. The Puechmaillemethod supports the presence of a single genetic cluster. This method appears to be more adapted to sparsely described environmental and demographic sampling (Puechmaille 2016). This finding (i.e., a single cluster including all individuals) aligns with other studies on Pinnidae using multiple genetic markers like *A. pectina* in the Yellow Sea and East China Sea (An et al. 2012; Xue et al. 2014; Zhu et al. 2024) or *P. nobilis* in the Mediterranean Sea (Peyran et al. 2022; Sarafidou et al. 2023).

Based on a total sample of 1389 individuals and an estimated size of the total population ranking between 1800 and 2000 individuals based on the sampling strategy, the observed effective population size (*N*_e_) computed using COLONY (*N*_e_ of 902 individuals [814–994]) thus represents 50% of the census size. This unusual ratio *N*_e_/*N* suggests a relatively equal reproductive contribution among individuals. This unusually high *N*_e_/*N* ratio, compared to values typically observed in marine organisms, often ranging from 0.001 to 0.1 (Frankham 1995; Palstra and Ruzzante 2008), suggests low reproductive skew and supports the hypothesis of a well‐mixed and genetically stable population.

### 4.4 Identified Family Structure and Relatedness Patterns

Based on relatedness values, we identified 299 distinct family clusters using the UPGMA method, encompassing 97% of the individuals sampled. This extensive family structuring strongly supports the high effective population size (*N*_e_) estimated from genetic data and confirms that a large proportion of the population actively contributes to reproduction.

This result is further confirmed by the COLONY analysis, which, despite reconstructing fewer families, still revealed a substantial number of natural kinship clusters (similar to those identified with the UPGMA approach). Detecting such a broad and balanced family structure involving most of the population is uncommon in marine bivalves. No single family dominates the population structure. The largest family identified through UPGMA contained only 19 individuals, a small fraction of the total effective population, indicating that family formation is common and not restricted to a few reproductive events, consistent with the limited presence of small‐sized individuals (i.e., juveniles) in the population.

The reproductive dynamics observed in this study, notably the high Ne/N ratio, contrast with patterns documented in many marine bivalves. In these species, high fecundity combined with substantial larval mortality frequently leads to “sweepstakes reproductive success”, resulting in low *N*_e_/*N* ratios (Hedgecock and Pudovkin 2011). Under a “sweepstakes reproductive success” model, only a small fraction of individuals successfully contribute to the next generation due to stochastic variation in larval survival. Several empirical studies illustrate this skewed reproductive output. For instance, in the *P. nobilis* from Peyrefite Bay (France), a single family composed of 67 individuals dominated the population structure, reflecting a strong reproductive inequality (Peyran et al. 2022). Similar reproductive imbalance patterns have also been reported in *Crassostrea gigas* (Boudry et al. 2002) and *Pecten maximus* (Morvezen et al. 2013), further exemplifying how variance in reproductive success can drastically reduce Ne in marine bivalves.

Taken together, these results suggest that when recruitment does occur, it likely involves a large number of breeders contributing at the same time, pointing toward occasional but collective reproductive events probably shaped by environmental conditions.

### 4.5 Spatial Distribution of Related Individuals

Although no clear global spatial structure was detected across the lagoon, we observed strong kinship aggregation among highly related individuals (*R* > 0.75), suggesting a localized recruitment mechanism. However, the majority of family members were more widely distributed, indicating that larval dispersal is not strictly limited and that multiple recruitment patterns may coexist.

The absence of juveniles in our samples is unlikely to result from detection bias, as small individuals have been successfully identified by the same divers during similar surveys conducted on another island. Given the presence of large, spatially coherent family clusters, this strongly suggests that recruitment events in *A. vexillum* are infrequent and occur within narrow temporal windows, during which only a subset of individuals successfully reproduce. Such episodic recruitment is consistent with strong environmental constraints during the larval or settlement phases, as shown in *A. pectinata* , where low salinity reduces larval survival (Kurihara et al. 2018). In addition, juvenile bivalves often require specific sediment conditions, such as documented for *P. nobilis* (Davenport et al. 2011), further influencing the settlement success.

Despite the critical importance of reproductive timing and environmental triggers, no study to date has documented the reproductive cycle of *A. vexillum* in French Polynesia. The timing of gonad maturation, spawning events, and associated environmental conditions (e.g., temperature, food availability, or particulate matter concentration) remain unknown. However, in closely related species such as *P. nobilis*, reproduction typically follows a seasonal cycle with spawning triggered by rising water temperatures (Deudero et al. 2017). By analogy, it is plausible that *A. vexillum* also exhibits seasonally driven reproductive cycles, although this remains to be demonstrated.

The spatial and genetic patterns we observed can be explained by three non‐mutually exclusive scenarios:

1. *Oceanic larval dispersal followed by lagoonal re‐entry*. In this scenario, typical of many bivalves, larvae develop outside the lagoon and only a fraction reenter the Bora‐Bora lagoon. Some settle elsewhere and are therefore not represented in our dataset. The overall genetic homogenization and the lack of strong spatial structure in the lagoon support this scenario. This model has already been already proposed in other bivalves of the region, such as *P. margaritifera* , within the same archipelago (Reisser et al. 2019).
2. *Local larval retention and recruitment near parental locations*. This scenario implies that the larvae develop and settle within the same lagoon. This hypothesis is strongly supported by our data, particularly the observed kinship aggregation and the numerous reconstructed families. This dynamic, common in enclosed environments, has been documented in *P. nobilis* in the Balearic Islands (Nebot‐Colomer et al. 2022), and suggested experimentally in *Tridacna squamosa*, where post‐settlement aggregative behavior was observed in juveniles under seminatural conditions in Singapore, where aggregative behavior during settlement has been reported (Huang et al. 2007). While philopatric behavior has not been shown in bivalves such as *T. maxima* in semi‐enclosed atolls in French Polynesia (Riquet et al. 2023), local retention remains the most consistent explanation for our findings.

As previously mentioned, these scenarios are not mutually exclusive, and it is likely that a combination of these processes, rather than a single one, shapes the observed genetic and spatial patterns.

### 4.6 Conservation Implication

A key finding of this study is the identification of family clusters encompassing nearly all sampled individuals, indicating that the *A. vexillum* population in Bora‐Bora is largely self‐renewing. This pattern strongly suggests that local reproduction and recruitment are the primary drivers of population persistence. Consequently, conservation strategies must prioritize local‐scale protection to sustain this self‐sustaining dynamic. Preserving the current population is essential, as it appears to serve as its own main recruitment source. The observation of kinship aggregation among closely related individuals further reinforces the need for spatially targeted conservation efforts, particularly focusing on high‐density zones (e.g., Northeast quadrant of Bora‐Bora lagoon) which likely function as recruitment hotspots. These zones should be considered as priority conservation areas.

Given the poorly documented life cycle and slow growth of *A. vexillum* , coupled with increasing anthropogenic pressures in the lagoon (e.g., coastal development, boating, pollution), the protection of benthic habitats emerges as a critical conservation priority. Moreover, protecting *A. vexillum* also supports the broader lagoon ecosystem, as this species associates with other organisms such as *Anchistus custoides* (Idris et al. 2022) or *Paranchistus ornatus* (Bruce 1977), as well as others poorly studied symbiotic or commensal species. The increased environmental complexity provided by the valves is sufficient to justify the unique ecological services that *A. vexillum* enhances habitat value and biodiversity.

The spawning phenomenon would greatly benefit from being linked to environmental signals. Although no study has documented food availability patterns in the lagoon of Bora‐Bora, seasonal and interannual variations have been observed in other Polynesian lagoons. For instance, Charpy and Charpy‐Roubaud (1991) reported fluctuations in particulate organic matter in Tikehau Atoll, including marked increases following episodic events such as cyclones. Similarly, Vollbrecht et al. (2021) identified persistent seasonal enrichment in chlorophyll‐a concentrations (i.e., Island Mass Effect) in Rangiroa Atoll, with fluctuations reflecting monsoonal and oceanographic regimes. These studies highlight the influence of seasonal environmental drivers on primary productivity and food availability in lagoon systems, reinforcing the need for ecological monitoring in Bora‐Bora to assess how such variability might affect reproductive success in sessile marine species.

Finally, implementing a long‐term genetic monitoring program would offer critical insights into population dynamics and conservation effectiveness. Building upon the comprehensive genetic baseline established in this study and the absence of new cohorts, such monitoring could help confirm the hypotheses on episodic recruitment and track future changes in recruitment success and family structure. It would also serve as a valuable tool to assess whether conservation interventions or environmental changes are influencing the population's ability to regenerate.

## Author Contributions

**Thomas Guttierez:** conceptualization (equal), data curation (lead), formal analysis (equal), investigation (lead), methodology (equal), resources (equal), software (lead), visualization (lead), writing – original draft (lead), writing – review and editing (equal). **Emilie Boissin:** conceptualization (equal), data curation (supporting), formal analysis (equal), investigation (supporting), methodology (equal), resources (equal), software (supporting), supervision (equal), validation (equal), writing – original draft (supporting), writing – review and editing (equal). **Serge Planes:** conceptualization (equal), formal analysis (equal), funding acquisition (lead), investigation (supporting), methodology (equal), project administration (lead), resources (equal), supervision (equal), validation (equal), writing – original draft (supporting), writing – review and editing (equal).

## Disclosure

Permission to Reproduce Material: No copyrighted material from third parties was used that required permission to reproduce.

## Ethics Statement

This research involved non‐lethal sampling of *A. vexillum*. All fieldwork was conducted under permits delivered by the local government of French Polynesia (DIREN), in compliance with national biodiversity regulations and with no impact on protected marine areas.

## Conflicts of Interest

The authors declare no conflicts of interest.

## Supporting information


**Table S1:** This table presents the genetic parameters of the microsatellite loci calculated over the 1205 individuals sampled in Bora‐Bora.
**Figure S1:** Bayesian clustering plots from structure and visualized with structure Selector.

## Data Availability

All microsatellite genotype data and metadata associated with this study are archived in GenBank as required by journal policy (Microsatellite sequences: PP586094–PP586098, PP586102, PP586104–PP586105, PP586108, PP586110, PP586112–PP586115, PP586117–PP586119, PP586121–PP586125, PP586128, PP586132–PP586133, PP586138–PP586139, PP586141–PP586143). The complete multilocus genotype matrix of the 1389 individuals analyzed is archived on Zenodo and accessible at the following https://doi.org/10.5281/zenodo.15685718. All data have been made available to editors and reviewers during the peer‐review process.
