# Supplementary material for: Genetic Relatedness and Parentage Analysis as a Framework to Enhance Local Conservation Strategies for Marine Species
Source: Ecol Evol. 2025 Sep 16;15(9):e72184. doi: 10.1002/ece3.72184 (PMC12439284; doi:10.1002/ece3.72184)
Supplement: Supplementary file 1 — Table S1: This table presents the genetic parameters of the microsatellite loci calculated over the 1205 individuals sampled in Bora‐Bora. Figure S1: Bayesian clustering plots from structure and visualized with structure Selector. [file ECE3-15-e72184-s001.docx]

# Supplementary Materials

**Table S 1:** This table presents the genetic parameters of the microsatellite loci calculated over the 1205 individuals sampled in Bora-Bora. For each locus, the GenBank accession number, the amplification rate, the number of alleles per locus (N_a_), the inbreeding coefficient (F_IS_), and the observed (H_o_) and expected (Hₑ) heterozygosities are provided. The significance of F_is_ values was tested using Genetix with 10,000 permutations (p < 0.05: *, p < 0.01: **, p < 0.001: ***), while heterozygosity values were computed using GenAlEx.

| **Locus** | **GenBank accession N°** | **Amplification rate** | $\boldsymbol{N}_{\boldsymbol{a}}$ | $\boldsymbol{F}_{\boldsymbol{IS}}$ | $\boldsymbol{H}_{\boldsymbol{o}}$ | $\boldsymbol{H}_{\boldsymbol{e}}$ |
| --- | --- | --- | --- | --- | --- | --- |
| Av_AG_01 | PP586094 | 99% | 18 | 0,017 | 0,847 | 0,861 |
| Av_AG_02 | PP586095 | 99% | 17 | 0,004 | 0,825 | 0,828 |
| Av_AT_45 | PP586138 | 100% | 4 | *0,045 | 0,505 | 0,528 |
| Av_ATC_24 | PP586117 | 98% | 5 | -0,001 | 0,507 | 0,507 |
| Av_AAAT_30 | PP586123 | 98% | 8 | 0,034 | 0,275 | 0,285 |
| Av_AGAT_29 | PP586122 | 99% | 9 | **0,049 | 0,603 | 0,633 |
| Av_AAT_21 | PP586114 | 98% | 9 | 0,019 | 0,758 | 0,773 |
| Av_AAT_26 | PP586119 | 95% | 5 | 0,005 | 0,522 | 0,524 |
| Av_AC_09 | PP586102 | 99% | 12 | ***0,112 | 0,644 | 0,725 |
| Av_AC_11 | PP586104 | 97% | 13 | *0,024 | 0,741 | 0,759 |
| Av_AG_19 | PP586112 | 100% | 11 | *0,027 | 0,805 | 0,827 |
| Av_AC_46 | PP586139 | 100% | 6 | 0,004 | 0,303 | 0,304 |
| Av_AGAT_40 | PP586133 | 100% | 6 | -0,046 | 0,529 | 0,506 |
| Av_AAAT_20 | PP586113 | 97% | 6 | 0,027 | 0,575 | 0,590 |
| Av_AAAT_50 | PP586143 | 100% | 9 | **0,037 | 0,766 | 0,795 |
| Av_ACT_32 | PP586125 | 100% | 6 | -0,006 | 0,666 | 0,662 |
| Av_AG_03 | PP586096 | 100% | 21 | -0,006 | 0,911 | 0,905 |
| Av_AG_05 | PP586098 | 99% | 18 | 0,01 | 0,838 | 0,846 |
| Av_AAAT_48 | PP586141 | 98% | 7 | ***0,053 | 0,684 | 0,721 |
| Av_AAAT_49 | PP586142 | 97% | 6 | 0,024 | 0,586 | 0,601 |
| Av_ATC_12 | PP586105 | 100% | 12 | *0,024 | 0,796 | 0,815 |


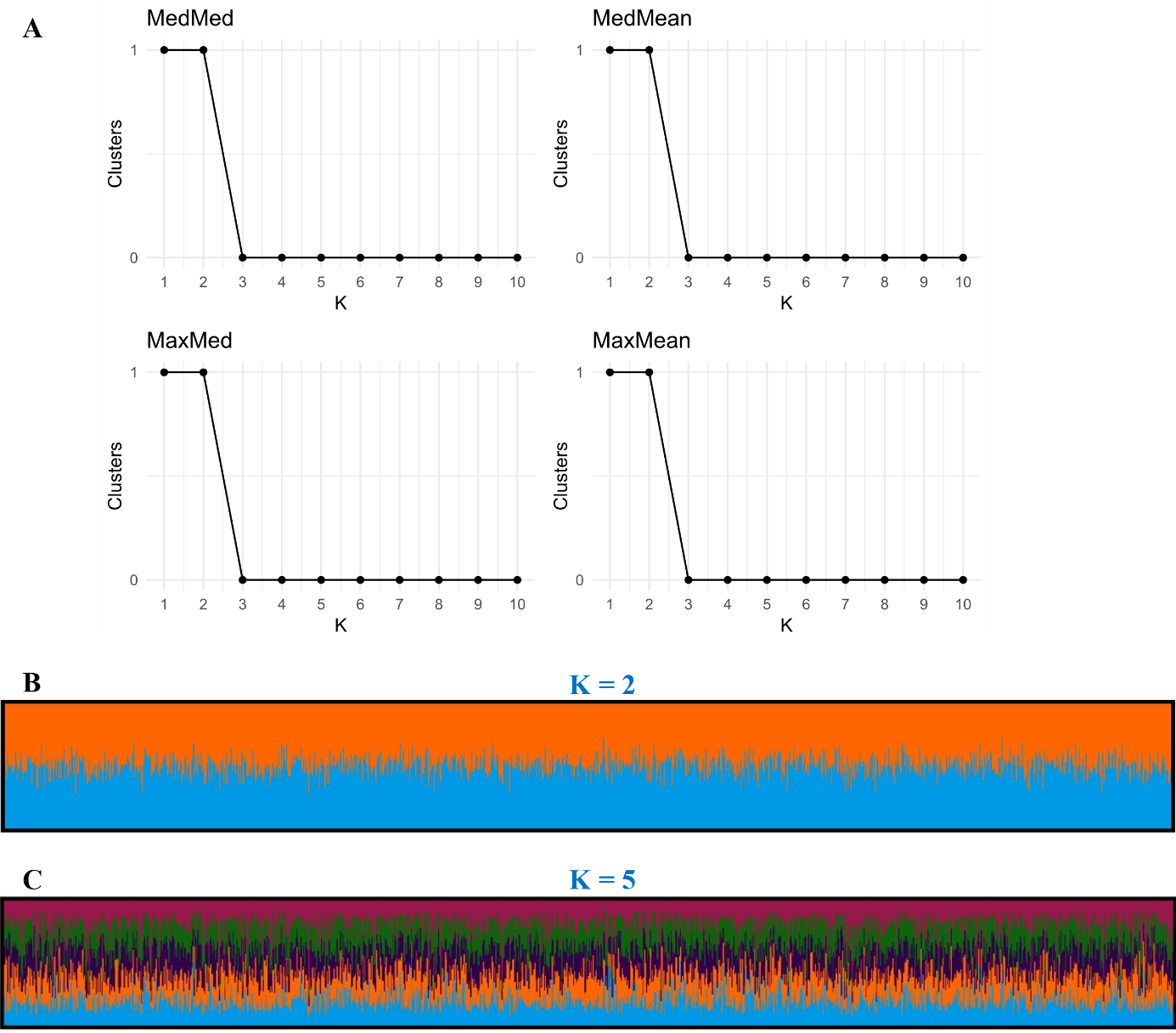


**Fig S.1**: Bayesian clustering plots from Structure and visualized with Structure Selector. The most likely number of K (cluster) was determined using the Puechmaille method (**A**), which excludes dissidents clusters as illustrated by the K=2 plot (**B**), while the Evanno method identifies K=5 (**C**)
